# Influence of Sample Processing on the Analysis of Carotenoids in Maize

**DOI:** 10.3390/molecules170911255

**Published:** 2012-09-21

**Authors:** Sol Rivera, Ramon Canela

**Affiliations:** Department of Chemistry, Lleida University, Alcalde Rovira Roure, 191, Lleida 25198, Spain

**Keywords:** carotenoids, extraction, solubility, injection solvent, liquid chromatography

## Abstract

We performed a number of tests with the aim to develop an effective extraction method for the analysis of carotenoid content in maize seed. Mixtures of methanol–ethyl acetate (6:4, v/v) and methanol–tetrahydrofuran (1:1, v/v) were the most effective solvent systems for carotenoid extraction from maize endosperm under the conditions assayed. In addition, we also addressed sample preparation prior to the analysis of carotenoids by liquid chromatography (LC). The LC response of extracted carotenoids and standards in several solvents was evaluated and results were related to the degree of solubility of these pigments. Three key factors were found to be important when selecting a suitable injection solvent: compatibility between the mobile phase and injection solvent, carotenoid polarity and content in the matrix.

## 1. Introduction

The choice of extraction methods for carotenoid analysis of food matrices is crucial because errors associated with the extraction process are potentially significant [1]. Given the wide variety of food products containing diverse carotenoids, there is no universally accepted or standard method for carotenoid extraction. The most widely accepted procedures involve extraction with organic solvents, including pentane, hexane, dichloromethane, chloroform, tetrahydrofuran (THF), methanol (MeOH), ethanol (EtOH), acetone, ethyl acetate, *n*-butanol, and petroleum ether [2,3,4,5,6]. Many techniques propose the use of freeze-dried material [7], a saponification step to hydrolyze carotenol esters, and the removal of lipids and chlorophylls, which may interfere with the chromatographic detection of carotenoids [4,5,8]. MeOH and THF are commonly used as first extraction solvents for maize seeds. Hexane, petroleum ether and ethyl ether are applied as second extraction solvents [9,10,11,12,13]. Although THF and ethyl ether are widely used because of their high capacity to solubilize carotenoids, such solvents can form peroxides, which can rapidly degrade carotenoids and may contribute to secondary products [14]. The addition of antioxidants, such as 2,6-bis(1,1-dimethylethyl)-4-methylphenol (BHT), to the solvent is therefore recommended [5]. In order to minimize auto-oxidation and *cis*-*trans* isomerization, carotenoid extraction must be carried out rapidly, avoiding exposure to light, oxygen, high temperatures and pro-oxidant metals, such as iron or copper [5].

Here we propose a new method for extracting carotenoids for maize and improved the performance of a chromatographic system which allows the separation of various carotenoids in less than 15 min. In addition, we provide information about the injection solvent and carotenoid concentrations recommended for this system.

## 2. Results and Discussion

### 2.1. Improvements in the Extraction Process

Initially, the method described by Naqvi [13] was used to extract carotenoids from maize endosperm. However, as this approach involves THF in the first step of the carotenoid extraction procedure (Section 3.3.1), we replaced it to prevent the formation of peroxides, which are known to catalyze carotenoid decomposition. Thus, we evaluated five modifications of the solvent system used to extract total carotenoids from maize seeds. *Modification 1* employed EtOH 100%; *modification 2*, acetone 100%; *modification 3*, acetone 100%, but after weighing the sample, it was covered with water (about 400 μL) and the mixture was allowed to stand at room temperature for 1 h before starting the extraction; *modification 4*, acetone–EtOH–hexane (1:1:2, v/v); and *modification 5*, MeOH–ethyl acetate (6:4, v/v). Table 1 shows the methods used for carotenoid extraction. Each extraction was replicated two or three times and the mean value of the total carotenoid content was used as the Output Factor. Higher values indicate greater extraction efficiency. The total carotenoid content was calculated spectrophotometrically using the following equation [15]:



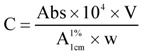
(1)


where A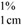 = absorption coefficient, which is defined as the theoretical absorbance of a solution of 1% (w/v) concentration (*i.e.*, g in 100 mL) in a cuvette with a path length of 1 cm. Lutein and zeaxanthin are the major carotenoids in maize. Therefore, an average value for A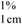 of 2,332 was used [15]. C = total carotenoid content (μg/g) in a given sample on dry weight basis. Abs = absorbance measured at 450 nm. V = volume (mL). W = weight of sample (g). 10^4^ = conversion factor to obtain the concentration in units of μg/g.

The total carotenoid content obtained with the six methods ranged from 106.5 to 142.1 μg/g DW. The percentage of non-extracted carotenoids was calculated by assigning a value of 100% to the method that resulted in the highest total carotenoids extracted (Table 1).

**Table 1:** Total carotenoids extracted from maize endosperm with the different solvent systems.

Modified method	Solvents	Ref.	Replicates	Output Factor ^c^ (μg/g DW)	% Non-extracted carotenoids
1	EtOH	[ 16,17]	2	111.8 ± 7.10	21.3
2	Acetone	[ 18,19]	3	106.5 ± 2.61	25.0
3	Acetone ^a^	[ 20]	3	126.3 ± 2.50	11.10
4	Acetone–EtOH–hexane (1:1:2, v/v)	[ 21]	2	120.9 ± 3.64	14.9
5	MeOH–ethyl–acetate (6:4, v/v) ^b^	-	3	141.6 ± 2.12	0
Reference	MeOH–THF (1:1, v/v)	[ 13,22,23]	3	142.1 ± 1.94	0

^a^ Samples were hydrated; ^b^ This mixture of solvents was developed in our laboratory; ^c^ Results are presented as means ± standard deviation (SD) from the same sample batch.

The solvents used to replace THF were selected considering various factors. MeOH and EtOH were tested because they affect cell wall permeability. This feature is relevant because carotenoids are confined to plant cells and the walls of these cells are complex in terms of chemical composition. We tested mixtures of MeOH, EtOH, acetone (polar solvents), ethyl acetate (medium-polar solvent) and hexane, a non-polar solvent, to search for the co-solubilization of carotenoids with different polarities in the samples. Non-polar carotenoids (e.g., lycopene and *β*-carotene) are more soluble in hexane and ethyl acetate [24,25] while more polar carotenoids (e.g., lutein or epoxy carotenoids) show greater solubility in EtOH and acetone [6,26]. Because acetone is widely used for carotenoid extraction [5], we assayed it as the first extraction solvent to replace THF.

The reference method and *modification 5* (using THF-MeOH 1:1, v/v and MeOH–ethyl acetate 6:4, v/v as solvents, respectively) were the most effective in extracting carotenoids from maize endosperm. The total extracted carotenoids for these two approaches was 142.1 and 141.6 μg/g DW, respectively. Indeed, only with these methods was a complete loss of color observed in the samples (from yellow to white), thereby indicating a suitable extraction capacity. A Student’s *t*-test determined that there was no statistically significant difference between the total carotenoid content obtained with the two methods (*t* calculated value: 0.27 < *t* critical value: 2.78 for 4 degrees of freedom at *α* = 0.05). *Modification 3* resulted in a higher total content of extracted carotenoid than *modification 4*, followed by *modifications 1* and *2*. The total carotenoids extracted with these methods were 126.3, 120.9, 111.8 and 106.5 μg/g DW, respectively.

The Student’s *t*-test showed a statistically significant difference (*t* calculated value: 12.38 > *t* critical value: 2.78 for four degrees of freedom at *α* = 0.05) between the total carotenoid content obtained in *modifications 2* and *3*. Consequently, it could be concluded that the degree of hydration of the samples accounts for the differences observed in the amount of carotenoids extracted with acetone. Although the presence of water could decrease carotenoid solubility in the extraction solvent, the higher carotenoid content achieved using *modification 3* might be attributable to the fact that water enhanced acetone penetration of the endosperm, thus increasing the extractability of the carotenoids [20]. Hence, the degree of sample hydration influences the choice of solvents used to extract carotenoids efficiently and reproducibly [6,27].

Howe *et al.* compared several procedures to extract maize kernels [7]. Among those examined, that described by Kurilich and Juvik [28] was found to be the most reliable method to determine the content of carotenoids in maize. The method requires the saponification of the sample with 80% potassium hydroxide w/v at 85 °C before extraction of carotenoids with hexane. Although carotenoids in maize are generally not present in the ester form [7], saponification was performed to remove saponifiable lipids, which could interfere with chromatographic analysis. Contrary to Howe *et al.* [7], our procedures did not require a saponification step since the embryo was removed to eliminate the presence of lipids. These results demonstrate the relevance of initial sample preparation prior to extraction. *Modification 5* was chosen to carry out the carotenoid extraction in the further analyses, since the reference method included THF.

#### 2.1.1. Effect of Adding BHT to the Extraction Solvents

In order to establish whether the addition of BHT to the extraction solvents used in *modification 5* favored carotenoid stability during analysis, transgenic maize TM2 was extracted with and without BHT. Samples were injected into a chromatograph 48 h after extraction. There was no statistically significant difference between the individual and total carotenoid content in samples in spite of using an anti-oxidant (Table 2). Indeed, the *t* calculated value was always lower than the *t* critical value for all cases. These results demonstrate that addition of BHT to the extraction solvents can be omitted provided that carotenoids are analyzed within 48 h of storage at −80 °C.

**Table 2:** Comparison of the individual and total carotenoid content of samples extracted with and without BHT.

Carotenoid	With BHT (μg/g DW)	Without BHT (μg/g DW)	*t* Calculated	*t* Critical	Degree of freedom ^a^
Astaxanthin	8.18 ± 0.06	8.22 ± 0.06	0.76	2.78	4
Adonixanthin	2.46 ± 0.03	2.51 ± 0.11	0.80	2.78	4
Zeax+lut	2.25 ± 0.11	2.37 ± 0.36	0.56	2.78	4
Adonirubin	1.52 ± 0.02	1.52 ± 0.01	0.36	2.78	4
Canthaxanthin	0.98 ± 0.00	0.96 ± 0.05	0.47	2.78	4
Total carotenoids	15.39 ± 0.16	15.84 ± 0.58	1.29	2.78	4

^a^ *α* = 0.05; abbreviations: Zeax+lut, sum of the concentrations of zeaxanthin and lutein; total carotenoids, total carotenoid content.

### 2.2. Solubility of Carotenoids

In a previous study [29], we developed a chromatographic system using ultra high performance liquid chromatography (UHPLC), in which 16 carotenoids were separated in less than 15 min. We used this chromatographic system here to separate carotenoids extracted from maize. However, further improvements were made to the system. Hence, the solvent used to dissolve the extracted carotenoids and standards, and the mobile phase were studied.

#### 2.2.1. Solubility of the Carotenoids Extracted from Maize in the Injection Solvent

Most carotenoids are insoluble in water and soluble in organic solvents such as acetone, alcohol, THF, ethyl ether, chloroform and ethyl acetate [27]. Nevertheless, their solubility depends on the presence of different functional groups. To ensure the complete solubilization of these pigments and to avoid incompatibility of the injection solvent with the mobile phase, combinations of acetone and 2-propanol (*i*PrOH) with the mobile phase (only solvent A, ACN–MeOH 7:3, v/v) were tested as injection solvent. Acetone and *i*PrOH were selected because they are miscible with the mobile phase and less polar than ACN and MeOH. Therefore, it was considered that these solvents might contribute to increasing the miscibility of carotenes. Solvent A was included as one of the components of the injection solvent since it is advisable to prepare the sample in the operating mobile phase for the best peak shape and sensitivity [30]. Various aliquots of the same maize sample (TM1) were obtained. Each one was dissolved in the same volume of injection solvent. The injection volume was also identical in all cases. This approach facilitated comparison between the corresponding chromatographic peaks and hence evaluation of the effect of the various injection solvents. 

Table 3 shows the injection solvents used to dissolve the carotenoids in TM1. No variations in retention time were observed when samples were dissolved in the different injection solvents (data not shown). The highest concentrations of pigments were obtained using a mixture of mobile phase–acetone 6.7:3.3 v/v rather than mobile phase alone as the injection solvent. For example, with this mixture the content obtained for zeaxanthin and lutein was 4.49 μg/g DW while with mobile phase alone this concentration dropped to 3.89 μg/g DW. A reduced percentage of acetone (sample dissolved in mobile phase–acetone 7.5:2.5 v/v) led to a decrease in the content of all carotenoids (Table 3). With this injection solvent, a concentration of 4.20 μg/g DW was obtained for zeaxanthin and lutein. The sample dissolved in ACN–MeOH–*i*PrOH 8.5:1:0.5 v/v/v did not show any increase in carotenoid contents (3.05 μg/g DW was obtained for zeaxanthin and lutein). Nevertheless, the lowest content of carotenoids were obtained dissolving the sample in 100% acetone. Acetone produced 2.20 μg/g DW of zeaxanthin and lutein content. Therefore, our results indicated that combinations of solvents were more advantageous to improve sample solubility than a single solvent such as acetone. 

**Table 3:** Effect of the injection solvent on the determination of the final carotenoid content of transgenic maize. A homogeneous lyophilizedmaize sample (TM1) was used for all experiments.

Injection solvent ^a^	Anther μg/g DW	Adonix μg/g DW	Lut+zeax μg/g DW	*α*-Crypt μg/g DW
Acetone 100%	0.12	1.93	2.20	0.98
Mobile phase ^b^–acetone 6.7:3.3, v/v	0.24	4.71	4.49	1.36
Mobile phase ^b^ 100%	0.20	4.48	3.89	1.27
Mobile phase ^b^–acetone 7.5:2.5, v/v	0.22	4.39	4.20	1.26
ACN–MeOH– *i*PrOH 8.5:1:0.5, v/v/v	0.15	3.31	3.05	1.12

^a^ Chromatographic conditions are described in Section 3.3.5; ^b^ Solvent A, ACN: MeOH 7:3, v/v. Abbreviations: Anther, antheraxanthin; Adonix, adonixanthin; Lut, lutein; Zeax, zeaxanthin; *α*-Crypt, *α*-cryptoxanthin.

**Figure 1:**
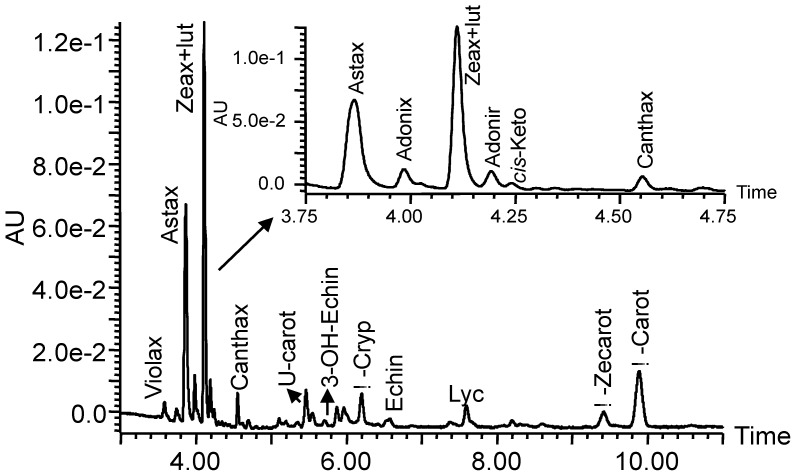
Carotenoid profile of the transgenic maize line TM2. Abbreviations: Violax, violaxanthin; Astax, astaxanthin; Zeax, zeaxanthin; Lut, lutein; Adonix, adonixanthin; Adonir, adonirubin; *cis*-Keto, *cis*-unknown ketocarotenoid; Canthax, canthaxanthin; U-cart, unknown carotenoid; 3-OH-Echinen, 3-OH-echinenone; *β*-Cryp, *β*-cryptoxanthin; Echin, echinenone; Lyc, lycopene; *β*-Zeacarot, *β*-zeacarotene, *β*-Carot, *β*-carotene.

Nevertheless, acetone was required to increase the solubility of the sample under the specific chromatographic conditions used because of the polarity of carotenoids present in TM1 (antheraxanthin, adonixanthin, lutein, zeaxanthin and *α*-cryptoxanthin). Thus, the mixture of mobile phase–acetone 6.7:3.3, v/v was chosen as the injection solvent. Figure 1 shows the separation of a mixture of carotenoids in the transgenic maize line TM2 using the improved UHPLC system.

#### 2.2.2. Preparation of the Carotenoid Standards

The solvents used to dissolve the carotenoid standards were chosen considering either the previously reported carotenoid solubility or the availability of the absorption coefficient of each pigment.

Stock carotenoid solutions were prepared in EtOH, acetone and hexane [31]. Carotenoid concentrations were determined spectrophotometrically. Table 4 shows the solvent and the value of A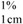 used to quantify each pigment. Working solutions were prepared from stock solutions by sampling an aliquot and diluting it with injection solvent. Solution concentrations were assessed by UHPLC analysis. For those carotenoids dissolved in hexane (canthaxanthin, *β*-cryptoxanthin, *β*-carotene, lycopene and phytoene), working solutions were prepared from stock solutions by evaporating an aliquot under nitrogen and diluting it with injection solvent. Calibration curves were obtained from peak area by injecting mixtures of standards. Table 5 shows the linear regression data for each carotenoid standard curve.

**Table 4:** Concentrations of carotenoid stock solutions used to build calibration curves.

Carotenoid	Solvent	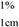	Stock carotenoid concentration (μg/mL)
*cis*-Neoxanthin	EtOH	2,380	19.64 ^a^
Violaxanthin	EtOH	2,550	16.26 ^a^
Antheraxanthin	EtOH	2,350	17.53 ^a^
Astaxanthin	EtOH	2,100	1.55 ^a^ and 4.96 ^b^
Astaxanthin	Injection solvent	-	5.12 ^b^
Zeaxanthin	Acetone	2,340	32.31 ^a^
Lutein	EtOH	2,550	21.57 ^a^
Canthaxanthin	Hexane	2,200	0.27 ^a^ and 4.53 ^b^
Canthaxanthin	Injection solvent	-	5.70 ^b^
*β*-Cryptoxanthin	Hexane	2,400	35.00 ^a^
*β*-Carotene	Hexane	2,590	24.85 ^a^
Lycopene	Hexane	3,450	8.26 ^a^
*cis*-Phytoene	Hexane	915	16.16 ^a^

^a^ Concentration was determined spectrophotometrically; ^b^ Concentration was determined by dividing the mass of the carotenoid by the total volume of solution.

**Table 5:** Linear regression data obtained for several carotenoid standard curves under UHPLC conditions.

Carotenoid	Linear range (μg/mL)	Slope	Intercept	R ^2^
*cis*-Neoxanthin	0.04−19.64	2,481 ± 7.57	−136.62 ± 16.28	0.9999
Violaxanthin	0.03−16.26	2,516 ± 5.59	−121.93 ± 12.97	0.9994
Antheraxanthin	0.03−15.53	2,509 ± 22.13	−413.83 ± 4.13	0.9970
Astaxanthin	0.04−5.12	1,825 ± 6.43	−50.37 ± 10.51	0.9999
Astaxanthin ^a^	−	14,149	4414.1	0.7741
Lutein	0.02−17.25	2,475 ± 81.74	−626.20 ± 35.78	0.9952
Zeaxanthin	0.03−17.23	2,578 ± 38.04	−86.96 ± 25.8	0.9996
Canthaxanthin	0.02−5.70	1,787 ± 4.24	−43.96 ± 16.13	0.9995
Canthaxanthin ^b^	−	9,613	1583.1	0.9320
*β*-Cryptoxanthin	0.04−18.67	2,379 ± 0.35	−444.17 ± 31.46	0.9988
Lycopene	0.3−3.11	1,398 ± 104.40	−121.72 ± 24.88	0.9998
*β*-Carotene	0.1−24.85	1,484 ± 27.22	−189.02 ± 29.80	0.9998
*cis*-Phytoene	0.08−16.16	1,990 ± 285.46	−259.82 ± 37.32	0.9989

^a^ Unknown linear range due to the solvent used to prepare the stock solution (EtOH) not being completely soluble; ^b^ Unknown linear range due to the solvent used to prepare the stock solution (hexane) not being completely soluble.

Solubilization problems were encountered for carotenes (when lycopene and *β*-carotene were dissolved in hexane) and for ketocarotenoids (when astaxanthin and canthaxanthin were dissolved in EtOH and hexane respectively). Chloroform, dichloromethane, hexane, ethyl acetate and THF [6,24,32,33] are known to dissolve lycopene and *β*-carotene. Thus, we chose hexane to solubilize these compounds. Initially, we attempted to prepare 100 μg/mL stock solutions of lycopene and *β*-carotene in hexane, but a precipitate was observed in the bottom of the vessels. Consequently, to ensure that carotenes were completely dissolved, stock solutions of carotenes were prepared again in hexane but in lower concentrations (Table 4). Thus, stock solutions of 24.85 μg/mL for *β*-carotene and 8.26 μg/mL for lycopene were prepared. We did not encounter solubilization problems with these concentrations. In addition, the calibration curves of these pigments (Table 5) indicated that the chromatographic peak areas of carotenes gave a linear plot throughout the concentration range studied.

Similarly, canthaxanthin and astaxanthin were not properly dissolved in hexane and EtOH respectively. Because of the problems of solubilization observed with these compounds, we determined and compared their concentrations using two different methods: dividing the mass of the carotenoid by the total volume of solution (theoretical concentration) and spectrophotometrically (experimental concentration).

The theoretical and experimental concentration obtained for canthaxanthin was 4.53 and 0.27 μg/mL, respectively whereas for astaxanthin it was 4.96 and 1.55 μg/mL, respectively. The lower concentrations of ketocarotenoids obtained experimentally indicated that hexane and EtOH were not appropriate solvents for canthaxanthin and astaxanthin, respectively. In addition, Table 5 shows that the calibration curves of these two pigments were characterized by a poor r-squared (R^2^ < 0.94). Therefore, we prepared stock ketocarotenoid solutions in the injection solvent of 5.12 μg/mL for astaxanthin and 5.70 μg/mL for canthaxanthin (Table 4). Table 5 shows that the calibration curves of these pigments dissolved in the injection solvent gave a linear plot throughout the concentration range studied. The lack of information about carotenoid absorption coefficients in a variety of organic solvents hampers the use of more appropriate solvents for ketocarotenoids.

We did not encounter any solubilization problems with the concentration range used for: (a) violaxanthin, antheraxanthin, neoxanthin and lutein, dissolved in EtOH; or (b) zeaxathin, dissolved in acetone and (c) *β*-cryptoxanthin, dissolved in hexane. As reported previously [26,27,34,35], oxygen-functionalized carotenoids showed satisfactory solubility in MeOH, EtOH and acetone.

Given the concentrations of carotenoids expected in maize samples, we did not prepare standard concentrations above 40 μg/mL. However, in our experience, higher concentrations of oxygen-functionalized carotenoids can be prepared with the injection solvent used here when needed. For example, concentrations of 100 μg/mL can be prepared for violaxanthin and neoxanthin. If higher carotene concentrations were required, changes in the injection solvent should be made to increase their solubility. For example, when lycopene is the target analyte, the following injection solvents have been used: chloroform 100%, to analyze extracts of tomato fruit pericarps [36]; ethyl acetate 100% [37] and *n*-butanol–ACN–dichloromethane (3:7:0.1, v/v/v) [38] to analyze extracts of tomato fruit.

Konings *et al.* [35] prepared stock solutions of lutein, zeaxanthin, *β*-carotene and lycopene with the same solvents used in this study. However, they used a mixture of MeOH–THF (7.5:2.5, v/v) as injection solvent. Under the chromatographic conditions applied, they observed a higher linear range for lutein, zeaxanthin and *β*-carotene than for lycopene. The smaller linearity range of lycopene (from 0 to 3.5 μg/mL) was explained by the lower solubility of this compound in the injection solvent. Nevertheless, the choice of the injection solvent was a compromise between satisfactory solubility of carotenoids, compatibility with the mobile phase, and the absence of peak distortions.

When carotenoid standard solutions are used several times and stored under N_2_ or Ar, their concentrations should be evaluated since the inert gas introduced various times into the vial evaporates the solvent, thereby changing the original carotenoid concentration. Thus, it is advisable to either divide the volume of carotenoid standard solutions into vials, putting only the volume required for each analysis into single vials, or to dry the standard solutions and redissolve these in each analysis. In addition, attention should be paid when many carotenoid standards at high concentrations are solubilized in the same solvent as some might precipitate. Thus, it is preferable to prepare various mixtures of carotenoids to ensure the complete solubilization of all analytes.

## 3. Experimental

### 3.1. Chemicals

*β*-Carotene, lycopene, lutein, *β*-cryptoxanthin, astaxanthin were purchased from Sigma-Aldrich Fine Chemicals (St. Louis, MO, USA). Canthaxanthin and zeaxanthin were acquired from Fluka (Buchs SG, Switzerland). Phytoene, violaxanthin, neoxanthin, and antheraxanthin were purchased from Carotenature (Lupsingen, Switzerland). EtOH, *i*PrOH, MeOH, ethyl acetate, hexane, ethyl eter, THF, ACN and acetone (HPLC grade purity) were supplied by J.T. Baker (Deventer, The Netherlands). Water was prepared using a Milli-Q reagent water system. 

### 3.2. Plant Material

The maize plants were generated by combinatorial nuclear transformation, as reported in Zhu *et al.* [39]. Transgenic maize lines TM1 and TM2, expressing several carotenogenic genes, were selected to optimize the extraction process and the chromatographic system.

### 3.3. Methods

#### 3.3.1. Reference Method

To protect carotenoids from degradation and oxidation, the extraction was conducted under limited light and THF was treated with sodium metal to remove peroxides. The procedure described by Naqvi [13] was used as reference method. Maize endosperm was excised by removing the seed coat and embryo. Only the maize endosperm was used for extraction because carotenoids were designed to accumulate only in this tissue by virtue of the genetic construct used to create the transgenic maize plants (endosperm-specific expression) [39]. Samples were freeze-dried and ground into a fine powder using a mortar and pestle. Maize seeds were lyophilized in order to prevent either microbial or chemical deterioration, which may be caused by the water content in the matrix. In addition, we chose to work with dried rather than fresh material because the former is more amenable to small-scale analysis and more easily homogenized. 50 or 100 [40] mg of sample was extracted with 15 mL of MeOH–THF (1:1, v/v) at 60 °C for 20 min and this mixture was continuously shaken. It was then put on ice until it reached room temperature and the liquid phase was filtered into a separatory funnel (if the residue exhibited color after extraction, it was re-extracted with 5 mL of the first extraction solvent at 60 °C for 5 min and the second extract was combined with the first one). 15 mL of hexane–diethyl ether (9:1, v/v) was added to the organic extract and the mixture was shaken vigorously. Then, 20 mL of saturated sodium chloride solution was added and the mixture was shaken again. The aqueous phase was removed and the organic phase was washed with water once again. The organic phase was concentrated under N_2_ at 37 °C until the volume was adjusted to 5 mL. 1 mL of blank solution was transferred to a cuvette and this was used to set the baseline absorbance of the spectrophotometer at 450 nm. The absorbance of 1 mL of the organic phase was determined. This organic phase was returned to the tube and left under N_2_ for further drying. When the sample was completely dry, Ar was flushed into the vial and carotenoids were stored at −80 °C until LC analysis. 

No saponification step was included because carotenoids are generally not present in ester forms in maize [7]. This step also has the inherent disadvantage of causing carotenoid losses [12].

When carotenoids are the only pigments present in the samples, extraction is facilitated as the process can be monitored. Consequently, loss of color was used as an indication of complete or satisfactory carotenoid extraction from the matrix.

#### 3.3.2. Blank Solution

A blank solution was prepared by carrying out the same extraction process as described in Section 3.3.1, without sample.

#### 3.3.3. Extraction Using BHT

BHT at a concentration of 0.1% was added to the extraction solvents used in *modification 5*: MeOH–ethyl acetate 6:4, v/v and hexane–diethyl ether 9:1, v/v. Each extraction was carried out in triplicate.

#### 3.3.4. Chromatographic Analysis

UHPLC-PDA analysis was carried out using an ACQUITY Ultra Performance LC^TM^ system linked to a photodiode array (PDA) 2996 detector (Waters, Milford, MA, USA). MassLynx^TM^ software version 4.1 (Waters) was used to control the instruments, and for data acquisition and processing. UHPLC chromatographic separation was performed on a reversed-phase column ACQUITY UPLC^®^ BEH 130Å C18, 1.7 μm, 2.1 × 100 mm (Waters) and a gradient system with the mobile phase consisting of solvent A, ACN–MeOH: (7:3, v/v) and solvent B, H_2_O 100%. The gradient program used is shown in Table 6. Column and sample temperatures were set at 32 °C and 25 °C respectively. Before use, all solutions were passed through Millex 0.2-μm nylon membrane syringe filters (Millipore, Bedford, MA, USA). The injection volume was 5 μL.

**Table 6:** Gradient profile used in the chromatographic separation of carotenoids.

Time (min)	Flow Rate (mL/min)	A (%, v/v)	B (%, v/v)	Curve
Initial	0.4	85	15	Linear
2.0	0.4	85	15	Linear
3.0	0.4	100	0	Linear
7.0	0.4	100	0	Linear
8.0	0.6	100	0	Linear
11.6	0.6	100	0	Linear
12.6	0.4	85	15	Linear
15.0	0.4	85	15	Linear

### 3.4. Ultraviolet and Visible (UV-vis) Spectroscopy

Absorption spectra and absorbance were recorded using a UNICAM UV/VIS Spectrometer UV2 ATI (Cambridge, UK).

### 3.5. Statistical Analysis

The Student’s *t*-test was used to determine differences in the mean values of carotenoid content obtained by the extraction methods. Microsoft Excel version 2010 (Microsoft Corp.) was used for data analysis.

## 4. Conclusions

Several factors, such as the polarity of the carotenoids present in the sample, sample preparation before extraction, and the chemical form of carotenoids in a given sample matrix (free form or bound to other compounds), should be considered in order to develop the most efficient extraction method. The reference method and *modification 5* showed the best performance at extracting carotenoids from maize endosperm. As the former involves THF, *modification 5* was chosen to carry out carotenoid extraction. This approach circumvents the presence of peroxides in the extraction solvents. The extraction method developed proved to be relatively fast for the determination of carotenoid pigments in maize endosperm. Furthermore, it allowed the simultaneous determination of various carotenoids in the samples. The injection solvent should be chosen on the basis of its compatibility with the mobile phase, and the polarity and concentrations of the carotenoids in the matrix. Thus, we recommend that chromatographic systems be adapted to suit the particular carotenoid profile being analyzed.
